# *catena*-Poly[[[aqua­copper(II)]bis­[μ-bis(3,5-dimethyl-1*H*-pyrazol-4-yl) selenide]] bis­(tetra­fluorido­borate) bis­(triphenyl­phosphine oxide) monohydrate]

**DOI:** 10.1107/S1600536810012997

**Published:** 2010-04-17

**Authors:** Maksym Seredyuk, Kateryna O. Znovjyak, Yurii S. Moroz, Vadim A. Pavlenko, Igor O. Fritsky

**Affiliations:** a Department of Chemistry, National Taras Shevchenko University, Volodymyrska Street 64, 01601 Kyiv, Ukraine

## Abstract

The title compound, {[Cu(C_10_H_14_N_4_Se)_2_(H_2_O)](BF_4_)_2_·2C_18_H_15_PO·H_2_O}_*n*_, has a polymeric structure where each Cu^II^ ion adopts a square-pyramidal coordination constituted by four N atoms of pyrazole moieties in the equatorial plane and an axial O atom of a water mol­ecule. A pair of bis­(3,5-dimethyl-1*H*-pyrazol-4-yl) selenide ligands bridges the Cu^II^ centres into a chain extending along the *c* axis. The water mol­ecules, anions and triphenyl­phosphine oxide mol­ecules are involved in inter­molecular hydrogen bonding, which links the chains into a three-dimensional network.

## Related literature

For general background, see: Farha *et al.* (2009 ▶); Shibahara *et al.* (2007 ▶); Zhang *et al.* (2009 ▶). For related structures, see: Seredyuk *et al.* (2007 ▶, 2009 ▶).
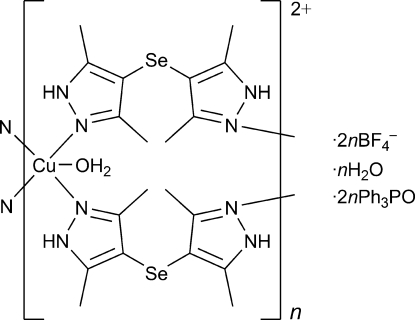


## Experimental

### 

#### Crystal data

[Cu(C_10_H_14_N_4_Se)_2_(H_2_O)](BF_4_)_2_·2C_18_H_15_OP·H_2_O
*M* *_r_* = 1386.17
Monoclinic, 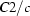

*a* = 21.4560 (4) Å
*b* = 15.3590 (4) Å
*c* = 18.4910 (6) Å
β = 97.74 (2)°
*V* = 6038.0 (3) Å^3^
*Z* = 4
Mo *K*α radiation
μ = 1.70 mm^−1^
*T* = 100 K
0.09 × 0.07 × 0.04 mm

#### Data collection

Kuma KM4 CCD area-detector diffractometer
34362 measured reflections
6876 independent reflections
6210 reflections with *I* > 2σ(*I*)
*R*_int_ = 0.045

#### Refinement

*R*[*F* ^2^ > 2σ(*F* ^2^)] = 0.043
*wR*(*F* ^2^) = 0.082
*S* = 1.16
6876 reflections
384 parameters
H-atom parameters constrained
Δρ_max_ = 0.63 e Å^−3^
Δρ_min_ = −0.38 e Å^−3^

### 

Data collection: *KM-4-CCD* (Kuma, 1999 ▶); cell refinement: *KM-4-CCD*; data reduction: *KM-4-CCD*; program(s) used to solve structure: *SHELXS97* (Sheldrick, 2008 ▶); program(s) used to refine structure: *SHELXL97* (Sheldrick, 2008 ▶); molecular graphics: *ORTEP-3 for Windows* (Farrugia, 1997 ▶); software used to prepare material for publication: *WinGX* (Farrugia, 1999 ▶).

## Supplementary Material

Crystal structure: contains datablocks I, global. DOI: 10.1107/S1600536810012997/ds2023sup1.cif

Structure factors: contains datablocks I. DOI: 10.1107/S1600536810012997/ds2023Isup2.hkl

Additional supplementary materials:  crystallographic information; 3D view; checkCIF report

## Figures and Tables

**Table 1:** Hydrogen-bond geometry (Å, °)

*D*—H⋯*A*	*D*—H	H⋯*A*	*D*⋯*A*	*D*—H⋯*A*
O1*W*—H1*W*⋯F2	0.90	2.14	3.002 (3)	161
O1*W*—H1*W*⋯F3	0.90	2.51	3.115 (3)	125
O1*W*—H2*W*⋯O2	0.95	1.90	2.785 (3)	155
O1—H1O⋯O2	0.81	1.95	2.752 (2)	173
N1—H1N⋯F4^i^	0.88	2.06	2.861 (3)	152
N4—H4N⋯O1*W*^ii^	0.88	1.86	2.730 (3)	170

Symmetry codes: (i) 
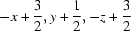
; (ii) 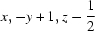.

**Fig. 1.**
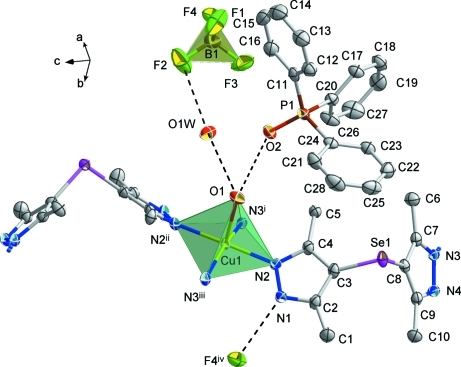
A portion of the crystal structure of the title compound showing the labeling scheme and 80% probability displacement ellipsoids [(i) 1.5 - x, 1.5 - y, 1 - z; (ii) 1.5 - x, 0.5 + y, 1.5 - z; (iii) -0.5 + x, 1.5 - y, 0.5 + z; (iv) 1 - x, 1 + y, 1.5 - z]. Hydrogen bonds are indicated by dashed lines. H atoms are omitted for clarity.

**Fig. 2.**
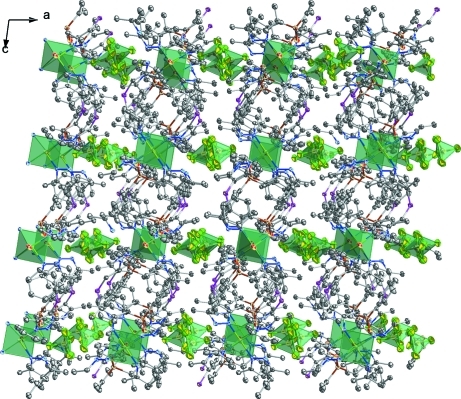
A packing diagram of the title compound. H atoms are omitted for clarity.

## supplementary crystallographic information

### Comment

Study of organometallic polymers is a well elaborated research area in coordination chemistry. Infinite molecular polymeric arrays are potentially applicable as specifically ordered crystalline substances with reversible selective sorption (Farha *et al.*, 2009; Zhang *et al.*, 2009), electrical conductivity (Zhang *et al.*, 2009) and molecular magnetism functionality (Shibahara *et al.*, 2007).

The title compound, [Cu(H_2_O)(C_10_H_14_N_4_Se)_2_][(BF_4_)_2_.(Ph_3_PO)_2_.H_2_O, was prepared in a water–methanolic medium by mixing solutions of Cu(BF_4_)_2_.6H_2_O and the mixture of the ligand bis(3,5-dimethyl-1*H*-pyrazolyl)selenide (L) and trisphenylphosphine oxide. It has similar structure to the copper compounds reported recently (Seredyuk *et al.*, 2007, 2009). A pyramidal environment of the Cu^II^ ion is constituted by four non-coplanar N atoms of pyrazolyl cycles (distances Cu—N are 1.997 (2) and 2.040 (2) Å, distance Cu—O is 2.222 (2) Å). Symmetrically equivalent ligand molecules in *cis*-bonding configuration are linked to Cu^II^ ion in a double-stranded bridge fashion (Fig. 1). Formed one-dimensional linear chain is running along the *c* axis where each Cu atom deviates from the average position by a value of ±0.279 (0) Å (Fig. 2). One of the pyrazole cycles of the ligand molecule is involved in hydrogen bonding with F atom of the tetrafluoroborate anion (N···F 2.861 (3) Å) which additionally forms a hydrogen bond with the free water molecule (F···OW 3.002 (3) Å). Further, the coordinated water molecule is connected through hydrogen bonds with the free water molecule (O···OW 2.785 (3) Å) and the Ph_3_PO molecule (O···O 3.115 (3) Å).

### Experimental

Bis(3,5-dimethyl-1*H*-pyrazolyl)selenide was prepared according to early reported method (Seredyuk *et al.*, 2007). Copper(II) tetrafluoroborate hexahydrate (0.065 g, 0.19 mmol) in water (5 ml) was added to 5 ml of hot methanol solution of the ligand (0.100 g, 0.37 mmol) and thisphenylphosphine oxide (0.052 g, 0.19 mmol). After several days green crystals of the title compound suitable for X-ray analysis were isolated. Found: C 49.06, H 4.43, N 8.22; C_56_H_62_B_2_CuF_8_N_8_O_4_P_2_Se_2_ requires: C 49.16, H 4.57, N 8.19.

### Refinement

The H atoms were located from the difference Fourier map and were constrained to ride on their parent atoms with U_iso_ = 1.2–1.5U_eq_(parent atom). The highest peak is located 0.90 Å from atom F3 and the deepest hole is located 0.67 Å from atom F2.

### Crystal data

**Table:**

[Cu(C_10_H_14_N_4_Se)_2_(H_2_O)](BF_4_)_2_·2C_18_H_15_OP·H_2_O	*F*(000) = 2820
*M**_r_* = 1386.17	*D*_x_ = 1.525 Mg m^−^^3^
Monoclinic, *C*2/*c*	Mo *K*α radiation, λ = 0.71073 Å
Hall symbol: -C 2yc	Cell parameters from 34362 reflections
*a* = 21.4560 (4) Å	θ = 3.1–28.5°
*b* = 15.3590 (4) Å	µ = 1.70 mm^−^^1^
*c* = 18.4910 (6) Å	*T* = 100 K
β = 97.74 (2)°	Needle, green
*V* = 6038.0 (3) Å^3^	0.09 × 0.07 × 0.04 mm
*Z* = 4	

### Data collection

**Table:**

Kuma KM4 CCD area-detector diffractometer	6210 reflections with *I* > 2σ(*I*)
Radiation source: fine-focus sealed tube	*R*_int_ = 0.045
graphite	θ_max_ = 27.5°, θ_min_ = 3.1°
ω scans	*h* = −27→27
34362 measured reflections	*k* = −19→18
6876 independent reflections	*l* = −24→22

### Refinement

**Table:**

Refinement on *F*^2^	Primary atom site location: structure-invariant direct methods
Least-squares matrix: full	Secondary atom site location: difference Fourier map
*R*[*F*^2^ > 2σ(*F*^2^)] = 0.043	Hydrogen site location: inferred from neighbouring sites
*wR*(*F*^2^) = 0.082	H-atom parameters constrained
*S* = 1.16	*w* = 1/[σ^2^(*F*_o_^2^) + (0.0323*P*)^2^ + 9.8106*P*] where *P* = (*F*_o_^2^ + 2*F*_c_^2^)/3
6876 reflections	(Δ/σ)_max_ = 0.002
384 parameters	Δρ_max_ = 0.63 e Å^−^^3^
0 restraints	Δρ_min_ = −0.38 e Å^−^^3^

### Special details

**Table:**

Experimental. The H atoms were located from the difference Fourier map and were constrained to ride on their parent atoms with U_iso_ = 1.2–1.5U_eq_(parent atom). The highest peak is located 0.90 Å from atom F3 and the deepest hole is located 0.67 Å from atom F2.
Geometry. All s.u.'s (except the s.u. in the dihedral angle between two l.s. planes) are estimated using the full covariance matrix. The cell s.u.'s are taken into account individually in the estimation of s.u.'s in distances, angles and torsion angles; correlations between s.u.'s in cell parameters are only used when they are defined by crystal symmetry. An approximate (isotropic) treatment of cell s.u.'s is used for estimating s.u.'s involving l.s. planes.
Refinement. Refinement of *F*^2^ against ALL reflections. The weighted *R*-factor wR and goodness of fit *S* are based on *F*^2^, conventional *R*-factors *R* are based on *F*, with *F* set to zero for negative *F*^2^. The threshold expression of *F*^2^ > 2σ(*F*^2^) is used only for calculating *R*-factors(gt) etc. and is not relevant to the choice of reflections for refinement. *R*-factors based on *F*^2^ are statistically about twice as large as those based on *F*, and *R*- factors based on ALL data will be even larger.

### Fractional atomic coordinates and isotropic or equivalent isotropic displacement parameters (Å^2^)

**Table:**

	*x*	*y*	*z*	*U*_iso_*/*U*_eq_	
C1	0.76965 (12)	0.52259 (18)	0.58609 (14)	0.0230 (6)	
H1A	0.7463	0.4882	0.6180	0.035*	
H1B	0.7520	0.5126	0.5351	0.035*	
H1C	0.7664	0.5845	0.5977	0.035*	
C2	0.83728 (11)	0.49578 (16)	0.59738 (12)	0.0161 (5)	
C3	0.87223 (11)	0.44383 (15)	0.55608 (12)	0.0150 (5)	
C4	0.93294 (11)	0.43713 (15)	0.59578 (12)	0.0140 (5)	
C5	0.98757 (11)	0.38947 (16)	0.57328 (13)	0.0163 (5)	
H5A	1.0132	0.4296	0.5485	0.025*	
H5B	0.9724	0.3421	0.5400	0.025*	
H5C	1.0131	0.3653	0.6165	0.025*	
C6	0.97077 (12)	0.35740 (16)	0.37061 (14)	0.0199 (5)	
H6A	0.9845	0.3431	0.3236	0.030*	
H6B	0.9494	0.3070	0.3885	0.030*	
H6C	1.0075	0.3725	0.4059	0.030*	
C7	0.92668 (11)	0.43281 (15)	0.36128 (12)	0.0142 (5)	
C8	0.87522 (11)	0.45220 (15)	0.39826 (12)	0.0134 (5)	
C9	0.84777 (11)	0.52685 (16)	0.36613 (12)	0.0156 (5)	
C10	0.78973 (12)	0.57463 (17)	0.37889 (14)	0.0207 (5)	
H10A	0.7855	0.6274	0.3489	0.031*	
H10B	0.7926	0.5906	0.4305	0.031*	
H10C	0.7530	0.5372	0.3656	0.031*	
C11	0.93216 (12)	0.04009 (16)	0.84170 (13)	0.0172 (5)	
C12	0.97655 (13)	−0.02714 (16)	0.84964 (13)	0.0204 (5)	
H12	1.0177	−0.0166	0.8740	0.024*	
C13	0.96033 (14)	−0.10957 (17)	0.82184 (15)	0.0264 (6)	
H13	0.9906	−0.1551	0.8269	0.032*	
C14	0.90039 (15)	−0.12532 (19)	0.78687 (15)	0.0326 (7)	
H14	0.8894	−0.1818	0.7683	0.039*	
C15	0.85628 (15)	−0.0592 (2)	0.77881 (15)	0.0328 (7)	
H15	0.8151	−0.0704	0.7547	0.039*	
C16	0.87171 (13)	0.02374 (18)	0.80581 (14)	0.0252 (6)	
H16	0.8413	0.0690	0.7999	0.030*	
C17	0.89479 (12)	0.09403 (16)	0.99831 (13)	0.0192 (5)	
H17	0.9065	0.0365	0.9869	0.023*	
C18	0.86575 (12)	0.10921 (18)	1.06015 (14)	0.0234 (6)	
H18	0.8580	0.0620	1.0909	0.028*	
C19	0.84825 (13)	0.19234 (19)	1.07704 (15)	0.0263 (6)	
H19	0.8285	0.2024	1.1193	0.032*	
C20	0.90673 (11)	0.16311 (15)	0.95319 (13)	0.0151 (5)	
C21	1.07652 (13)	0.14666 (17)	0.86366 (15)	0.0234 (6)	
H21	1.0631	0.1465	0.8126	0.028*	
C22	1.11652 (13)	0.14863 (17)	1.01317 (15)	0.0236 (6)	
H22	1.1304	0.1496	1.0642	0.028*	
C23	1.05261 (12)	0.14969 (16)	0.98780 (13)	0.0183 (5)	
H23	1.0228	0.1510	1.0215	0.022*	
C24	1.03194 (12)	0.14881 (15)	0.91255 (13)	0.0160 (5)	
C25	1.16016 (13)	0.14610 (17)	0.96428 (16)	0.0262 (6)	
H25	1.2038	0.1453	0.9819	0.031*	
C26	0.88851 (13)	0.24716 (17)	0.97001 (15)	0.0244 (6)	
H26	0.8959	0.2945	0.9392	0.029*	
C27	0.85963 (14)	0.26146 (19)	1.03183 (16)	0.0311 (7)	
H27	0.8476	0.3188	1.0434	0.037*	
C28	1.14020 (13)	0.14472 (19)	0.88991 (16)	0.0283 (6)	
H28	1.1703	0.1424	0.8567	0.034*	
N1	0.87644 (9)	0.51791 (13)	0.65756 (10)	0.0159 (4)	
H1N	0.8655	0.5510	0.6926	0.019*	
N2	0.93546 (9)	0.48303 (13)	0.65830 (10)	0.0132 (4)	
N3	0.93111 (9)	0.49421 (12)	0.30991 (10)	0.0130 (4)	
N4	0.88268 (9)	0.55081 (13)	0.31437 (10)	0.0140 (4)	
H4N	0.8752	0.5973	0.2867	0.017*	
O1	1.0000	0.33717 (15)	0.7500	0.0184 (5)	
H1O	0.9810	0.3047	0.7736	0.028*	
O2	0.93330 (8)	0.21702 (11)	0.81896 (9)	0.0200 (4)	
O1W	0.84524 (9)	0.31134 (11)	0.72590 (10)	0.0238 (4)	
H1W	0.8041	0.2973	0.7207	0.036*	
H2W	0.8687	0.2652	0.7505	0.036*	
Cu1	1.0000	0.48181 (3)	0.7500	0.01134 (9)	
Se1	0.841882 (11)	0.382203 (16)	0.468941 (12)	0.01567 (7)	
P1	0.94959 (3)	0.14841 (4)	0.87644 (3)	0.01450 (13)	
B1	0.71450 (15)	0.1759 (2)	0.75026 (17)	0.0261 (7)	
F4	0.65217 (8)	0.17089 (10)	0.76495 (9)	0.0292 (4)	
F3	0.75223 (9)	0.21024 (12)	0.81081 (10)	0.0423 (5)	
F1	0.73540 (9)	0.09284 (12)	0.73760 (10)	0.0459 (5)	
F2	0.71809 (9)	0.22853 (14)	0.68996 (10)	0.0503 (5)	

### Atomic displacement parameters (Å^2^)

**Table:**

	*U*^11^	*U*^22^	*U*^33^	*U*^12^	*U*^13^	*U*^23^
C1	0.0159 (13)	0.0333 (15)	0.0200 (13)	0.0052 (11)	0.0030 (10)	−0.0026 (11)
C2	0.0145 (12)	0.0212 (12)	0.0127 (11)	0.0000 (10)	0.0028 (9)	0.0022 (9)
C3	0.0155 (12)	0.0189 (12)	0.0111 (11)	−0.0018 (9)	0.0037 (9)	0.0021 (9)
C4	0.0172 (12)	0.0148 (11)	0.0107 (11)	−0.0012 (9)	0.0045 (9)	0.0027 (9)
C5	0.0153 (12)	0.0201 (12)	0.0134 (11)	0.0030 (10)	0.0014 (9)	−0.0025 (9)
C6	0.0209 (13)	0.0201 (13)	0.0197 (12)	0.0031 (10)	0.0064 (10)	0.0033 (10)
C7	0.0136 (12)	0.0179 (12)	0.0111 (11)	−0.0026 (9)	0.0016 (9)	−0.0020 (9)
C8	0.0115 (11)	0.0179 (12)	0.0107 (10)	−0.0023 (9)	0.0015 (9)	−0.0026 (9)
C9	0.0154 (12)	0.0197 (12)	0.0116 (11)	−0.0007 (10)	0.0019 (9)	−0.0018 (9)
C10	0.0175 (13)	0.0262 (14)	0.0191 (12)	0.0042 (10)	0.0045 (10)	−0.0011 (10)
C11	0.0211 (13)	0.0197 (12)	0.0118 (11)	−0.0025 (10)	0.0053 (9)	0.0012 (9)
C12	0.0236 (14)	0.0201 (13)	0.0184 (12)	−0.0010 (10)	0.0062 (10)	0.0004 (10)
C13	0.0363 (16)	0.0205 (13)	0.0245 (13)	−0.0015 (12)	0.0119 (12)	−0.0022 (11)
C14	0.048 (2)	0.0277 (15)	0.0234 (14)	−0.0133 (14)	0.0115 (13)	−0.0091 (12)
C15	0.0329 (17)	0.0432 (18)	0.0210 (14)	−0.0154 (14)	−0.0015 (12)	−0.0022 (12)
C16	0.0229 (14)	0.0309 (15)	0.0213 (13)	−0.0015 (11)	0.0017 (11)	0.0016 (11)
C17	0.0187 (13)	0.0180 (12)	0.0218 (13)	0.0002 (10)	0.0063 (10)	0.0014 (10)
C18	0.0212 (14)	0.0280 (14)	0.0223 (13)	−0.0012 (11)	0.0078 (11)	0.0063 (11)
C19	0.0238 (14)	0.0356 (16)	0.0217 (13)	−0.0007 (12)	0.0114 (11)	−0.0048 (11)
C20	0.0120 (11)	0.0172 (12)	0.0164 (11)	0.0012 (9)	0.0030 (9)	0.0016 (9)
C21	0.0234 (14)	0.0269 (14)	0.0213 (13)	0.0021 (11)	0.0084 (11)	0.0038 (11)
C22	0.0214 (14)	0.0227 (13)	0.0252 (14)	0.0010 (11)	−0.0020 (11)	0.0023 (11)
C23	0.0180 (13)	0.0175 (12)	0.0201 (12)	0.0005 (10)	0.0056 (10)	0.0008 (9)
C24	0.0166 (12)	0.0127 (11)	0.0194 (12)	0.0010 (9)	0.0045 (10)	0.0016 (9)
C25	0.0141 (13)	0.0231 (13)	0.0408 (16)	0.0011 (10)	0.0019 (11)	0.0076 (12)
C26	0.0254 (14)	0.0187 (13)	0.0318 (15)	0.0015 (11)	0.0140 (12)	0.0033 (11)
C27	0.0348 (17)	0.0221 (14)	0.0398 (17)	0.0035 (12)	0.0177 (14)	−0.0056 (12)
C28	0.0199 (14)	0.0318 (15)	0.0359 (16)	0.0044 (11)	0.0137 (12)	0.0100 (12)
N1	0.0150 (10)	0.0210 (10)	0.0121 (9)	0.0046 (8)	0.0032 (8)	−0.0002 (8)
N2	0.0113 (10)	0.0175 (10)	0.0113 (9)	0.0021 (8)	0.0029 (7)	0.0013 (7)
N3	0.0120 (10)	0.0157 (10)	0.0114 (9)	0.0017 (8)	0.0018 (7)	0.0006 (7)
N4	0.0138 (10)	0.0151 (10)	0.0135 (9)	0.0026 (8)	0.0028 (8)	0.0017 (7)
O1	0.0212 (13)	0.0162 (12)	0.0203 (12)	0.000	0.0111 (10)	0.000
O2	0.0196 (9)	0.0212 (9)	0.0195 (9)	0.0008 (7)	0.0038 (7)	0.0072 (7)
O1W	0.0204 (10)	0.0215 (9)	0.0283 (10)	−0.0019 (8)	−0.0006 (8)	−0.0022 (8)
Cu1	0.0110 (2)	0.0155 (2)	0.00792 (18)	0.000	0.00246 (14)	0.000
Se1	0.01537 (13)	0.02050 (13)	0.01152 (11)	−0.00501 (10)	0.00324 (8)	−0.00052 (9)
P1	0.0146 (3)	0.0151 (3)	0.0144 (3)	0.0005 (2)	0.0040 (2)	0.0027 (2)
B1	0.0247 (17)	0.0276 (16)	0.0271 (16)	−0.0012 (13)	0.0066 (13)	−0.0015 (13)
F4	0.0275 (9)	0.0238 (8)	0.0369 (9)	−0.0034 (7)	0.0066 (7)	0.0056 (7)
F3	0.0413 (11)	0.0443 (11)	0.0420 (10)	−0.0186 (9)	0.0080 (8)	−0.0179 (8)
F1	0.0429 (11)	0.0384 (10)	0.0501 (12)	0.0178 (9)	−0.0167 (9)	−0.0192 (9)
F2	0.0476 (12)	0.0625 (13)	0.0447 (11)	0.0072 (10)	0.0209 (9)	0.0234 (10)

### Geometric parameters (Å, °)

**Table:**

C1—C2	1.496 (3)	C17—H17	0.9500
C1—H1A	0.9800	C18—C19	1.378 (4)
C1—H1B	0.9800	C18—H18	0.9500
C1—H1C	0.9800	C19—C27	1.393 (4)
C2—N1	1.345 (3)	C19—H19	0.9500
C2—C3	1.392 (3)	C20—C26	1.396 (3)
C3—C4	1.410 (3)	C20—P1	1.806 (2)
C3—Se1	1.906 (2)	C21—C28	1.387 (4)
C4—N2	1.349 (3)	C21—C24	1.402 (3)
C4—C5	1.488 (3)	C21—H21	0.9500
C5—H5A	0.9800	C22—C25	1.387 (4)
C5—H5B	0.9800	C22—C23	1.388 (4)
C5—H5C	0.9800	C22—H22	0.9500
C6—C7	1.491 (3)	C23—C24	1.402 (3)
C6—H6A	0.9800	C23—H23	0.9500
C6—H6B	0.9800	C24—P1	1.803 (3)
C6—H6C	0.9800	C25—C28	1.384 (4)
C7—N3	1.351 (3)	C25—H25	0.9500
C7—C8	1.407 (3)	C26—C27	1.389 (4)
C8—C9	1.386 (3)	C26—H26	0.9500
C8—Se1	1.905 (2)	C27—H27	0.9500
C9—N4	1.344 (3)	C28—H28	0.9500
C9—C10	1.491 (3)	N1—N2	1.373 (3)
C10—H10A	0.9800	N1—H1N	0.8800
C10—H10B	0.9800	N2—Cu1	2.0397 (19)
C10—H10C	0.9800	N3—N4	1.366 (3)
C11—C16	1.398 (4)	N3—Cu1^i^	1.9971 (19)
C11—C12	1.399 (4)	N4—H4N	0.8800
C11—P1	1.804 (2)	O1—Cu1	2.222 (2)
C12—C13	1.393 (4)	O1—H1O	0.8082
C12—H12	0.9500	O2—P1	1.5040 (17)
C13—C14	1.381 (4)	O1W—H1W	0.9003
C13—H13	0.9500	O1W—H2W	0.9489
C14—C15	1.383 (4)	Cu1—N3^ii^	1.9971 (19)
C14—H14	0.9500	Cu1—N3^i^	1.9971 (19)
C15—C16	1.391 (4)	Cu1—N2^iii^	2.0397 (19)
C15—H15	0.9500	B1—F1	1.383 (4)
C16—H16	0.9500	B1—F2	1.387 (4)
C17—C18	1.394 (3)	B1—F3	1.394 (4)
C17—C20	1.395 (3)	B1—F4	1.402 (4)
			
C2—C1—H1A	109.5	C18—C19—H19	120.2
C2—C1—H1B	109.5	C27—C19—H19	120.2
H1A—C1—H1B	109.5	C17—C20—C26	119.4 (2)
C2—C1—H1C	109.5	C17—C20—P1	121.94 (18)
H1A—C1—H1C	109.5	C26—C20—P1	118.57 (18)
H1B—C1—H1C	109.5	C28—C21—C24	120.0 (2)
N1—C2—C3	106.0 (2)	C28—C21—H21	120.0
N1—C2—C1	122.2 (2)	C24—C21—H21	120.0
C3—C2—C1	131.7 (2)	C25—C22—C23	120.2 (2)
C2—C3—C4	106.5 (2)	C25—C22—H22	119.9
C2—C3—Se1	126.91 (18)	C23—C22—H22	119.9
C4—C3—Se1	126.17 (17)	C22—C23—C24	120.1 (2)
N2—C4—C3	109.6 (2)	C22—C23—H23	120.0
N2—C4—C5	123.7 (2)	C24—C23—H23	120.0
C3—C4—C5	126.7 (2)	C21—C24—C23	119.2 (2)
C4—C5—H5A	109.5	C21—C24—P1	118.74 (19)
C4—C5—H5B	109.5	C23—C24—P1	122.04 (19)
H5A—C5—H5B	109.5	C28—C25—C22	120.2 (3)
C4—C5—H5C	109.5	C28—C25—H25	119.9
H5A—C5—H5C	109.5	C22—C25—H25	119.9
H5B—C5—H5C	109.5	C27—C26—C20	119.9 (2)
C7—C6—H6A	109.5	C27—C26—H26	120.0
C7—C6—H6B	109.5	C20—C26—H26	120.0
H6A—C6—H6B	109.5	C26—C27—C19	120.5 (3)
C7—C6—H6C	109.5	C26—C27—H27	119.8
H6A—C6—H6C	109.5	C19—C27—H27	119.8
H6B—C6—H6C	109.5	C25—C28—C21	120.4 (2)
N3—C7—C8	109.2 (2)	C25—C28—H28	119.8
N3—C7—C6	121.3 (2)	C21—C28—H28	119.8
C8—C7—C6	129.5 (2)	C2—N1—N2	112.47 (19)
C9—C8—C7	106.4 (2)	C2—N1—H1N	123.8
C9—C8—Se1	125.73 (18)	N2—N1—H1N	123.8
C7—C8—Se1	127.30 (18)	C4—N2—N1	105.36 (18)
N4—C9—C8	106.6 (2)	C4—N2—Cu1	130.69 (16)
N4—C9—C10	122.4 (2)	N1—N2—Cu1	122.26 (14)
C8—C9—C10	130.9 (2)	C7—N3—N4	105.99 (18)
C9—C10—H10A	109.5	C7—N3—Cu1^i^	130.26 (16)
C9—C10—H10B	109.5	N4—N3—Cu1^i^	122.83 (14)
H10A—C10—H10B	109.5	C9—N4—N3	111.75 (19)
C9—C10—H10C	109.5	C9—N4—H4N	124.1
H10A—C10—H10C	109.5	N3—N4—H4N	124.1
H10B—C10—H10C	109.5	Cu1—O1—H1O	128.2
C16—C11—C12	119.5 (2)	H1W—O1W—H2W	108.7
C16—C11—P1	118.0 (2)	N3^ii^—Cu1—N3^i^	158.75 (11)
C12—C11—P1	122.53 (19)	N3^ii^—Cu1—N2	89.38 (8)
C13—C12—C11	120.0 (3)	N3^i^—Cu1—N2	90.43 (8)
C13—C12—H12	120.0	N3^ii^—Cu1—N2^iii^	90.43 (8)
C11—C12—H12	120.0	N3^i^—Cu1—N2^iii^	89.38 (8)
C14—C13—C12	120.2 (3)	N2—Cu1—N2^iii^	178.95 (11)
C14—C13—H13	119.9	N3^ii^—Cu1—O1	100.62 (6)
C12—C13—H13	119.9	N3^i^—Cu1—O1	100.62 (6)
C13—C14—C15	120.2 (3)	N2—Cu1—O1	90.53 (6)
C13—C14—H14	119.9	N2^iii^—Cu1—O1	90.53 (6)
C15—C14—H14	119.9	C8—Se1—C3	100.52 (10)
C14—C15—C16	120.5 (3)	O2—P1—C24	112.46 (11)
C14—C15—H15	119.7	O2—P1—C11	112.16 (11)
C16—C15—H15	119.7	C24—P1—C11	106.30 (11)
C15—C16—C11	119.7 (3)	O2—P1—C20	111.92 (11)
C15—C16—H16	120.2	C24—P1—C20	106.62 (11)
C11—C16—H16	120.2	C11—P1—C20	106.98 (11)
C18—C17—C20	120.1 (2)	F1—B1—F2	110.3 (2)
C18—C17—H17	119.9	F1—B1—F3	108.6 (3)
C20—C17—H17	119.9	F2—B1—F3	109.7 (3)
C19—C18—C17	120.4 (2)	F1—B1—F4	108.7 (2)
C19—C18—H18	119.8	F2—B1—F4	110.2 (2)
C17—C18—H18	119.8	F3—B1—F4	109.3 (2)
C18—C19—C27	119.7 (2)		
			
N1—C2—C3—C4	0.3 (3)	C3—C4—N2—N1	0.2 (2)
C1—C2—C3—C4	−176.6 (3)	C5—C4—N2—N1	178.7 (2)
N1—C2—C3—Se1	173.45 (17)	C3—C4—N2—Cu1	165.24 (16)
C1—C2—C3—Se1	−3.4 (4)	C5—C4—N2—Cu1	−16.2 (3)
C2—C3—C4—N2	−0.3 (3)	C2—N1—N2—C4	0.0 (3)
Se1—C3—C4—N2	−173.53 (16)	C2—N1—N2—Cu1	−166.62 (16)
C2—C3—C4—C5	−178.8 (2)	C8—C7—N3—N4	−0.5 (2)
Se1—C3—C4—C5	8.0 (3)	C6—C7—N3—N4	177.6 (2)
N3—C7—C8—C9	1.3 (3)	C8—C7—N3—Cu1^i^	168.54 (16)
C6—C7—C8—C9	−176.6 (2)	C6—C7—N3—Cu1^i^	−13.4 (3)
N3—C7—C8—Se1	173.23 (16)	C8—C9—N4—N3	1.3 (3)
C6—C7—C8—Se1	−4.7 (4)	C10—C9—N4—N3	−176.2 (2)
C7—C8—C9—N4	−1.6 (3)	C7—N3—N4—C9	−0.5 (2)
Se1—C8—C9—N4	−173.66 (16)	Cu1^i^—N3—N4—C9	−170.57 (15)
C7—C8—C9—C10	175.6 (2)	C4—N2—Cu1—N3^ii^	−146.4 (2)
Se1—C8—C9—C10	3.5 (4)	N1—N2—Cu1—N3^ii^	16.47 (17)
C16—C11—C12—C13	0.0 (4)	C4—N2—Cu1—N3^i^	54.8 (2)
P1—C11—C12—C13	179.83 (19)	N1—N2—Cu1—N3^i^	−142.28 (17)
C11—C12—C13—C14	−0.5 (4)	C4—N2—Cu1—O1	−45.8 (2)
C12—C13—C14—C15	0.5 (4)	N1—N2—Cu1—O1	117.09 (17)
C13—C14—C15—C16	−0.1 (4)	C9—C8—Se1—C3	−84.4 (2)
C14—C15—C16—C11	−0.4 (4)	C7—C8—Se1—C3	105.1 (2)
C12—C11—C16—C15	0.4 (4)	C2—C3—Se1—C8	106.5 (2)
P1—C11—C16—C15	−179.4 (2)	C4—C3—Se1—C8	−81.6 (2)
C20—C17—C18—C19	0.4 (4)	C21—C24—P1—O2	−51.1 (2)
C17—C18—C19—C27	0.1 (4)	C23—C24—P1—O2	130.0 (2)
C18—C17—C20—C26	−0.9 (4)	C21—C24—P1—C11	72.0 (2)
C18—C17—C20—P1	175.1 (2)	C23—C24—P1—C11	−106.8 (2)
C25—C22—C23—C24	−0.4 (4)	C21—C24—P1—C20	−174.10 (19)
C28—C21—C24—C23	0.5 (4)	C23—C24—P1—C20	7.0 (2)
C28—C21—C24—P1	−178.4 (2)	C16—C11—P1—O2	−52.4 (2)
C22—C23—C24—C21	0.2 (4)	C12—C11—P1—O2	127.8 (2)
C22—C23—C24—P1	179.02 (19)	C16—C11—P1—C24	−175.71 (19)
C23—C22—C25—C28	0.1 (4)	C12—C11—P1—C24	4.5 (2)
C17—C20—C26—C27	0.9 (4)	C16—C11—P1—C20	70.7 (2)
P1—C20—C26—C27	−175.2 (2)	C12—C11—P1—C20	−109.1 (2)
C20—C26—C27—C19	−0.5 (4)	C17—C20—P1—O2	155.2 (2)
C18—C19—C27—C26	−0.1 (5)	C26—C20—P1—O2	−28.8 (2)
C22—C25—C28—C21	0.6 (4)	C17—C20—P1—C24	−81.5 (2)
C24—C21—C28—C25	−0.9 (4)	C26—C20—P1—C24	94.6 (2)
C3—C2—N1—N2	−0.2 (3)	C17—C20—P1—C11	31.9 (2)
C1—C2—N1—N2	177.0 (2)	C26—C20—P1—C11	−152.0 (2)

Symmetry codes: (i) −*x*+2, −*y*+1, −*z*+1; (ii) *x*, −*y*+1, *z*+1/2; (iii) −*x*+2, *y*, −*z*+3/2.

### Hydrogen-bond geometry (Å, °)

**Table:**

*D*—H···*A*	*D*—H	H···*A*	*D*···*A*	*D*—H···*A*
O1W—H1W···F2	0.90	2.14	3.002 (3)	161
O1W—H1W···F3	0.90	2.51	3.115 (3)	125
O1W—H2W···O2	0.95	1.90	2.785 (3)	155
O1—H1O···O2	0.81	1.95	2.752 (2)	173
N1—H1N···F4^iv^	0.88	2.06	2.861 (3)	152
N4—H4N···O1W^v^	0.88	1.86	2.730 (3)	170

Symmetry codes: (iv) −*x*+3/2, *y*+1/2, −*z*+3/2; (v) *x*, −*y*+1, *z*−1/2.
